# Effectiveness of interprofessional education enhanced by live consultation observations for healthcare students and new professionals in Singapore: a retrospective cross-sectional study

**DOI:** 10.3352/jeehp.2025.22.21

**Published:** 2025-08-21

**Authors:** Lynette Mei Lim Goh, Wai Leong Chiu, Sky Wei Chee Koh

**Affiliations:** 1 National University Polyclinics, National University Health System, Singapore; 2 Yong Loo Lin School of Medicine, National University of Singapore, Singapore; The Catholic University of Korea, Korea

**Keywords:** Interprofessional education, Problem-based learning, Health occupations students, Patient care team, Communication, Singapore

## Abstract

This study aims to evaluate whether incorporating live consultation observations into interprofessional education (IPE) improves learning evaluation scores among healthcare professionals and students. A retrospective cross-sectional analysis was conducted using evaluation data from AHP IPE sessions held from January 2020 to December 2023 across 7 primary care clinics in Singapore. Evaluation scores were compared between sessions with facilitated discussions only (n=667) and sessions with additional live consultation observations (n=501). Logistic regression was used to analyze factors associated with perfect evaluation scores. Sessions that included live consultations were significantly more likely to achieve perfect evaluation scores (odds ratio [OR], 1.68; 95% confidence interval [CI], 1.27–2.22). Nursing/care coordinator and allied health professions (OR 2.07 and 1.76 respectively) were significantly more likely to give perfect scores compared to medical professions. Healthcare professionals were also more likely to give perfect scores than students (OR, 1.52; 95% CI,1.08–2.14), indicating enhanced perceived effectiveness. These findings support the use of experiential learning strategies to optimize interprofessional training outcomes.

## Graphical abstract

[Fig f1-jeehp-22-21]

## Background/rationale

Interprofessional collaboration is essential for effective healthcare delivery, enabling professionals from diverse disciplines to address complex patient needs and enhance quality of care [1]. The World Health Organization emphasizes the importance of interprofessional education (IPE) in fostering teamwork across healthcare disciplines, specialties and professions [2]. IPE occurs when “2 or more professionals learn with, from, and about each other,” promoting mutual understanding and improved communication through shared learning experiences. Through collaboration, healthcare professionals can provide more comprehensive and patient-centered care, ultimately leading to better health outcomes.

The benefits of IPE are well documented, including improvements in work ethic and mutual respect among healthcare professionals, as well as positive impacts on burnout, organizational climate, and culture [3]. Despite these benefits, the most effective learning modalities for IPE remain underexplored [4]. Many healthcare students and early-career professionals have limited exposure to the roles and responsibilities of other team members, which can hinder effective collaboration.

Traditional didactic sessions may not effectively bridge the gap between theory and practice, limiting the impact of IPE. Experiential learning approaches, such as live consultation observations, may offer more meaningful engagement and effectively bridge this gap [5]. These real-world experiences are considered valuable in IPE, allowing healthcare professionals and students to learn from each other and gain a deeper understanding of roles and responsibilities of other professions. However, few studies have directly compared the impact of live consultation observations versus standard discussions in IPE. To better understand the mechanisms by which these learning experiences facilitate professional growth, the experiential learning theory by Kolb [6] offers a helpful framework. According to Kolb [6], knowledge is constructed through a cyclical process of concrete experience, reflective observation, abstract conceptualization, and active experimentation. Facilitated sessions with live consultation observations enable participants to engage in this process by experiencing, reflecting upon, and conceptualizing collaborative care, which can subsequently influence their future practice.

## Objectives

This study aims to evaluate whether adding live consultation observations to standard facilitated discussions improves learning evaluation scores among healthcare professionals and students.

## Ethics statement

This study was exempt from ethics review as it involved anonymized, retrospective data and did not meet the criteria for human subject research. Approval was obtained from National University Polyclinics (NUP-RNR-2025-0011), and the study was conducted in accordance with the Declaration of Helsinki. Patient confidentiality and privacy were maintained through strict anonymization procedures and adherence to data protection regulations. A waiver of informed consent was granted, as the study was classified as presenting minimal risk to participants.

## Study design and setting

This retrospective cross-sectional study analyzed evaluation data from IPE sessions conducted by Allied Health Professionals (AHPs) at 7 large primary care clinics under the National University Polyclinics (NUP) in Singapore. These clinics serve approximately 2 million patient visits annually, with half related to chronic diseases [7]. NUP offers 6 allied health services: dietetics, financial counseling, medical social services, physiotherapy, podiatry, and psychology.

The study focused on IPE sessions held between January 1, 2020, and December 31, 2023, delivered as part of orientation programs for new healthcare professionals or as clinical training for healthcare students. Sessions took place at the 7 clinics based on participants’ placement or orientation schedules. No active recruitment was conducted, as the study utilized existing evaluation data collected retrospectively after each session.

Participants attended 1 of 2 IPE modalities for each allied health service, depending on patient appointment schedules and service availability: (1) Facilitated discussions involved structured, one-on-one sessions where allied health professionals shared their roles, patient cases, and approaches to care. These sessions provided opportunities for reflective learning and professional insight without direct patient interaction. (2) Facilitated discussions with live consultation observations included the same structured sessions as above, but also allowed participants to directly observe allied health professionals interacting with and treating patients. This provided active engagement and real-world exposure, supporting vicarious learning and reinforcing key principles of experiential learning.

Participants were allocated to attend at least 1 physical therapy service (podiatry or physiotherapy) and 1 counseling service (dietetics, medical social work, or psychology), depending on service availability. Each IPE session, whether with or without observation, lasted 60 to 120 minutes. The STROBE (Strengthening the Reporting of Observational Studies in Epidemiology) reporting guideline was used to structure the manuscript (Supplement 1).

## Participants

Eligible participants included healthcare students (medicine, nursing, pharmacy, and allied health) from local universities and polytechnics, as well as newly hired healthcare professionals at NUP who attended IPE sessions with AHPs. Inclusion required full attendance and completion of the post-session evaluation. To ensure anonymity, demographic data such as age, sex, and race were excluded due to identification risks, especially among professionals; only profession or student type was collected. Observers were assigned to professions other than their own to minimize evaluation bias and support IPE’s goal of cross-disciplinary understanding.

## Variables

Independent variables included profession type (medical, nursing or care coordinator, or allied health), occupation (healthcare professional or student), and type of AHP attached to (dietetics, financial counselling, medical social services, podiatry, psychology, or physiotherapy). The primary variable of interest was session type, categorized as either facilitated discussions or facilitated discussions with live consultation observation. The outcome variable was participant evaluation scores.

## Data sources/measurement

Participants completed an evaluation form developed by the NUP AHP educators’ workgroup, comprising educator leads from 6 services: dietetics, financial counselling, medical social work, physiotherapy, podiatry, and psychology. The instrument was developed collaboratively, drawing on the educator leads’ professional experience and understanding of interprofessional education needs. The form assessed session effectiveness across 4 domains using a 5-point Likert scale (1=strongly disagree to 5=strongly agree). Individual item scores were summed to produce an overall evaluation score, and mean scores were used to assess perceived training effectiveness. The 4 evaluation domains were: (1) Achievement of learning objectives: This section assessed whether the session successfully met participants’ desired learning goals. (2) Relevance and support: Participants evaluated the training’s relevance to clinical practice and the approachability of the AHP facilitator. (3) Educator’s knowledge and skills: This component measured the educator’s ability to impart knowledge and skills effectively. (4) Overall satisfaction: This component captured participants’ general impressions of session quality and impact.

## Study size

The study included all 352 participants attached to AHPs who attended a total of 1,168 IPE sessions and completed the evaluation forms, thereby capturing the entire population of interest and eliminating the need for a sample size calculation. We collected all participants’ responses and there were no missing data.

## Statistical methods

Preliminary analysis showed a skewed distribution of evaluation scores, with 63.5% of sessions receiving full marks (25 points). Chi-square tests were first used to assess associations between variables and full scores. To account for the skewness in evaluation scores, particularly the high proportion of perfect scores, a hurdle model was employed. This statistical approach is well-suited for analyzing count data with a large number of perfect scores, helping to mitigate potential analysis errors and provide more accurate insights. The multivariate hurdle model included logistic regression for the probability of full scores and a zero-truncated negative binomial model for non-full mark counts. Results were presented as odds ratios (OR) with 95% confidence intervals (CI) for the logistic component, and incidence rate ratios (IRR) with 95% CI for the count component. The analysis was performed in R ver. 4.4.2 (The R Foundation for Statistical Computing), with P<0.05 considered statistically significant.

## Main results

A total of 352 participants attached to AHPs completed 1,168 IPE sessions with evaluation forms, all of which were included in the study (Dataset 1). Of these sessions, 667 (57%) were facilitated discussions, and 501 (43%) included both facilitated discussions and live consultation observations (Table 1). The professional backgrounds of attendees were as follows: 45.5% medical, 38.1% nursing or care coordinator, and 16.5% allied health. The majority of participants (77.8%) were students. Medical social services accounted for the largest proportion of sessions (30.1%). The mean evaluation score was 23.7 (standard deviation=2.1), with a median of 25 (range, 15–25). Notably, 742 sessions (63.5%) received perfect scores (25/25).

Significant associations were found between session type and the likelihood of achieving perfect evaluation scores (P<0.001). Facilitated discussions with live consultation observations were more likely to receive perfect scores (OR, 1.73; 95% CI, 1.35–2.22) compared to facilitated discussions alone. Profession type was also significantly associated with perfect evaluation scores (P<0.001), with allied health having the highest proportion of perfect scores (71.0%) (Table 1). The type of AHP service was significantly associated with perfect evaluation scores (P=0.001), with podiatry sessions showing the highest proportion (81.6%). However, no significant association was found between occupation (student or healthcare professional) and evaluation scores (P=0.95).

In the multivariate analysis, the zero-truncated negative binomial model for non-full mark counts yielded non-significant results across all variables (Table 2). However, logistic regression identified significant associations with perfect evaluation scores. Facilitated discussions with live consultation observations were more likely to achieve perfect scores than facilitated discussions alone (OR, 1.68; 95% CI, 1.27–2.22). Nursing/care coordinators (OR, 2.07; 95% CI, 1.52–2.80) and allied health (OR, 1.76; 95% CI, 1.21–2.57) were more likely to give perfect scores compared to medical professionals. Healthcare professionals were also more likely to assign perfect scores than students (OR, 1.52; 95% CI, 1.08–2.14). Among allied health services, financial counselling participants were more likely to give perfect scores than those attached to dietetics (OR, 1.66; 95% CI, 1.08–2.54), with no significant differences found for other AHPs.

## Key results

The study aimed to evaluate the effectiveness of different IPE modalities and identify factors associated with high evaluation scores. Sessions incorporating live consultation observations were more likely to receive perfect scores (25/25) than sessions consisting of facilitated discussions alone. The participant’s profession type, occupation (student, staff), and attachment to financial counselling were significant predictors of perfect evaluation scores.

## Interpretation

The findings suggest that incorporating real-time observational components into IPE sessions was associated with higher rates of perfect evaluation scores and enhanced perceived satisfaction, likely due to greater contextual relevance and engagement. Observing interprofessional collaboration in real clinical settings may help learners connect theory to practice, potentially improving patient care and workload management.

Healthcare professionals rated IPE sessions more favorably than students, possibly reflecting greater relevance to their daily practice or differing expectations. Their clinical experience may lead to a heightened appreciation for collaborative practice, whereas students may lack sufficient exposure to fully understand its benefits.

Variations in satisfaction across professional subgroups may reflect differing engagement levels or familiarity with collaborative learning. Financial counsellors, for example, may have assigned higher scores due to their unique roles and perspectives in a primary care context. These findings underscore the importance of tailoring IPE design to the diverse roles, experiences, and expectations of healthcare professionals. The results support integrating authentic, observation-based learning, particularly for practicing professionals, and highlight the need for flexible, context-sensitive IPE approaches.

While the hurdle model was appropriate for addressing the statistical issue of perfect scores, the pronounced ceiling effect limits the model’s sensitivity to detect meaningful differences, as it does not account for underlying cultural biases that may inflate scores in our study.

## Comparison with previous studies

Our findings regarding the effectiveness of direct observation in IPE align with and expand upon previous research. While prior studies have emphasized the role of frequent, informal communication in fostering effective collaboration, our study contributes to the limited research on direct observation of collaborative practice. Consistent with existing literature, our results also showed that perceptions of IPE vary by professional role and clinical experience [8]. Notably, our study extends previous findings from student populations to working professionals [9], demonstrating the benefits of observation-based learning in both foundational and practice-based settings, and supporting research that highlights the positive learning value of live observations [10].

## Strengths

This study had several strengths, including a large, diverse sample of healthcare students and newly hired professionals. Conducting the study across 7 primary care clinics in Singapore enhanced its external validity and generalizability. Analyzing existing evaluation data minimized biases related to consent and recruitment. The use of a robust hurdle model addressed skewness in evaluation scores, while a standardized evaluation form developed by educator leads from each allied health profession ensured consistent assessment of session effectiveness.

## Limitations/generalizability

This study has several limitations. Non-randomized participant assignment may introduce selection bias, and the absence of demographic data limits understanding of influencing factors. Generalizability may be affected by the overrepresentation of medical students and the limited range of allied health professions. Unmeasured variables, such as prior experience, may confound outcomes. While the evaluation form was piloted within the workgroup to ensure clarity and relevance of questions, no formal reliability or validity testing was conducted, which may affect the robustness of the results. The use of Likert scales introduces potential bias, and scores may be influenced by social desirability or facilitator likability bias. Self-reported evaluations may not reflect actual changes in practice. Evaluations were collected anonymously immediately after the sessions to encourage honest feedback; however, this approach disallowed the study team from accounting for potential clustering, prevented linking responses to individual participants, and may have contributed to nonresponse bias. Participants who disliked the IPE session may have been less likely to submit the questionnaire, potentially resulting in nonresponse bias.

## Suggestions

Future research should improve methodology through randomized allocation, broader discipline representation, demographic data collection, and a mixed-methods approach that includes qualitative interviews and more nuanced open-ended feedback mechanisms. Testing alternative learning modalities can support scalable and accessible IPE. Long-term outcomes such as patient care, team collaboration, professional competency, and system efficiency should be assessed to guide the development of inclusive, effective programs for collaborative practice.

## Conclusion

While sessions incorporating real-time experiential components were associated with higher rates of perfect evaluation scores, the pronounced ceiling effect and potential for cultural or societal bias in scoring limit the ability to draw firm conclusions about true differences in perceived effectiveness or educational benefit. Our findings highlight the need for more sensitive feedback mechanisms and evaluation systems capable of capturing meaningful differences in learner experience and assessment of educational benefits. For example, future studies could use more granular rating scales (e.g., a 10-point instead of a 5-point scale) and adopt mixed-method approaches that combine quantitative ratings with qualitative feedback to provide a more comprehensive and nuanced understanding of participant experiences. Additionally, incorporating pre- and post-session measures of participants’ confidence in referring to allied health professionals could yield valuable insights into the practical impact of the education. This would allow researchers to assess both participants’ satisfaction with the sessions and tangible changes in their clinical decision-making confidence, offering a more robust measure of the program’s effectiveness in enhancing interdisciplinary care practices.

## Figures and Tables

**Figure f1-jeehp-22-21:**
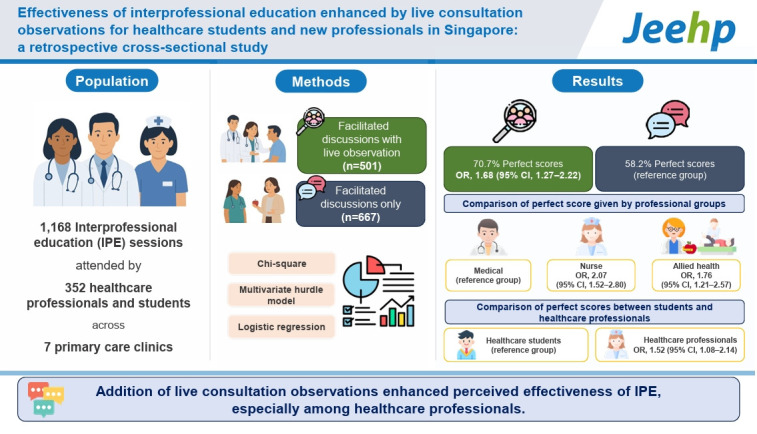


**Table 1.** Differences in evaluation scores between IPE modalities across services

Session characteristics	Overall	Perfect evaluation scores (25 points)	Not full scores (5–24 points)	P-value^a)^
Total	1,168 (100.0)	742 (63.5)	426 (36.5)	
Type of IPE				<0.001
Facilitated discussions with live consultation observation	501 (42.9)	354 (70.7)	147 (29.3)	
Facilitated discussions	667 (57.1)	388 (58.2)	279 (41.8)	
Profession type				<0.001
Allied health	193 (16.5)	137 (71.0)	56 (29.0)	
Nursing/care coordinator	445 (38.1)	243 (54.6)	202 (45.4)	
Medical	530 (45.4)	362 (68.3)	168 (31.7)	
Occupation				0.95
Healthcare professional	259 (22.2)	165 (63.7)	94 (36.3)	
Student	909 (77.8)	577 (63.5)	332 (36.5)	
Type of AHP attached to				0.001
Dietetics	279 (23.9)	179 (64.2)	100 (35.8)	
Financial counselling	181 (15.5)	130 (71.8)	51 (28.2)	
Medical social services	352 (30.1)	201 (57.1)	151 (42.9)	
Physiotherapy	232 (19.9)	149 (64.2)	83 (35.8)	
Podiatry	49 (4.2)	40 (81.6)	9 (18.4)	
Psychology	75 (6.4)	43 (57.3)	32 (42.7)	

Values are presented as number (%), unless otherwise stated. IPE, interprofessional education; AHP, Allied Health Professionals.

a) By chi-square test.

**Table 2.** Multivariate hurdle model analyzing factors affecting evaluation scores for IPE

Predictors	Zero-truncated negative binomial regression for evaluation scores (5–24)		Logistic regression for perfect evaluation score (25)	
	IRR	P-value	OR (95% CI)	P-value
Type of IPE				
Facilitated discussions only	1		1	
Facilitated discussions with live consultation observation	0.01	0.997	1.68 (1.27–2.22)	**<0.001**
Profession type				
Medical	1		1	
Nursing/care coordinator	0.01	0.995	2.07 (1.52–2.80)	**<0.001**
Allied health	0.15	0.996	1.76 (1.21–2.57)	**0.003**
Occupation				
Student	1		1	
Healthcare professional	0.11	0.996	1.52 (1.08–2.14)	**0.018**
Type of AHP attached to				
Dietetics	1		1	
Financial counselling	0.14	0.995	1.66 (1.08–2.54)	**0.02**
Medical social services	0.08	0.994	0.87 (0.62–1.22)	0.414
Physiotherapy	0.12	0.997	0.91 (0.63–1.32)	0.615
Podiatry	0.54	1.00	1.60 (0.73–3.50)	0.237
Psychology	0.52	0.999	0.85 (0.49–1.48)	0.568

Statistically significant results are marked in bold. IPE, interprofessional education; IRR, incidence rate ratio; OR, odds ratio; CI, confidence interval; AHP, Allied Health Professionals.

## Footnotes

**Data availability**
Data files are available from https://doi.org/10.7910/DVN/KRDOWB Dataset 1. Response data of questionnaire or survey responses.

## Supplementary materials

Supplement 1. STROBE checklist.

Supplement 2. Audio recording of the abstract.
