# Supplementary material for: Effectiveness of interprofessional education enhanced by live consultation observations for healthcare students and new professionals in Singapore: a retrospective cross-sectional study
Source: J Educ Eval Health Prof. 2025 Aug 21;22:21. doi: 10.3352/jeehp.2025.22.21 (PMC12768549; doi:10.3352/jeehp.2025.22.21)
Supplement: Supplementary file 2 — Supplement 1. STROBE checklist. [file jeehp-22-21-Suppl.docx]

STROBE Statement—checklist of items that should be included in reports of observational studies

|  | Item No. | Recommendation | Page  No. | Relevant text from manuscript |
| --- | --- | --- | --- | --- |
| **Title and abstract** | 1 | (*a*) Indicate the study’s design with a commonly used term in the title or the abstract | 1 | Comparative effectiveness of interprofessional education |
|  |  | (*b*) Provide in the abstract an informative and balanced summary of what was done and what was found | 1 | Incorporating live consultation observations into IPE sessions can enhance their effectiveness, tailored to certain professions and experience levels. |
| Introduction | | | |  |
| Background/rationale | 2 | Explain the scientific background and rationale for the investigation being reported | 2 | Interprofessional collaboration (IPE) is essential for effective healthcare delivery. Experiential learning approaches, such as live consultation observations, may offer more meaningful engagement. However, direct evidence supporting the impact of live consultation observations remains limited. |
| Objectives | 3 | State specific objectives, including any prespecified hypotheses | 2 | evaluate whether adding live consultation observations to standard facilitated discussions improves learning evaluation scores |
| Methods | | | |  |
| Study design | 4 | Present key elements of study design early in the paper | 3 | retrospective cross-sectional study analyzed evaluation data from IPE sessions |
| Setting | 5 | Describe the setting, locations, and relevant dates, including periods of recruitment, exposure, follow-up, and data collection |  | IPE sessions conducted by Allied Health Professionals (AHPs) at seven large primary care clinics; held between 1 January 2020 and 31 December 2023, delivered as part of orientation program for new healthcare professionals or clinical training for healthcare students. Sessions took place across the seven clinics based on participants’ placement or orientation schedules. |
| Participants | 6 | (*a*) ***Cross-sectional study*—Give the eligibility criteria, and the sources and methods of selection of participants** | 3 | No active recruitment was conducted, as the study utilized existing evaluation data collected retrospectively following each session. Participants attended one of two IPE modalities for each allied health service, depending on patient appointment availability and service availability. Eligible participants included healthcare students (medicine, nursing, pharmacy, and allied health) from local universities and polytechnics, and newly hired healthcare professionals at NUP who attended IPE sessions with AHPs. |
|  |  | (*b*) *Cohort study*—For matched studies, give matching criteria and number of exposed and unexposed  *Case-control study*—For matched studies, give matching criteria and the number of controls per case |  | NA |
| Variables | 7 | Clearly define all outcomes, exposures, predictors, potential confounders, and effect modifiers. Give diagnostic criteria, if applicable | 3-4 | The primary outcomes were evaluation scores of the interprofessional education (IPE) sessions, measured using a standardized evaluation form with scores ranging from 5 to 25. Exposure variables included the type of IPE modality—facilitated discussions only versus facilitated discussions with live consultation observations. Predictors included profession type and occupation. Potential confounders such as prior experience were not measured. No diagnostic criteria were applicable. |
| Data sources/ measurement | 8* | For each variable of interest, give sources of data and details of methods of assessment (measurement). Describe comparability of assessment methods if there is more than one group | 3-4 | Evaluation scores were collected using a standardized evaluation form developed by educator leads from each allied health profession. The form assesses participants’ perceptions of the IPE sessions on a scale of 5–25.  The main exposure—the IPE delivery method (facilitated discussion only vs facilitated discussion with live consultation observation)—was documented by the study team. Profession and occupation were recorded based on participant self-identification.  The standardized evaluation form was used across both groups, ensuring comparability of assessment methods. |
| Bias | 9 | Describe any efforts to address potential sources of bias | 8 | To minimize potential bias, we used a standardized evaluation form across both groups and trained educators to facilitate sessions in a consistent manner. Analyzing existing evaluation data also helped reduce biases related to consent and recruitment. Nonetheless, nonresponse bias may remain, as participants who chose not to submit their questionnaires may differ from those who did. |
| Study size | 10 | Explain how the study size was arrived at | 5 | We included all available evaluation forms from participants who attended the IPE sessions across the seven primary care clinics during the study period. Our study size was determined by the response rate from participants, with 1168 evaluation forms analyzed. |

Continued on next page

| Quantitative variables | 11 | Explain how quantitative variables were handled in the analyses. If applicable, describe which groupings were chosen and why | 4-5 | Quantitative variables like evaluation scores were analyzed as continuous and categorical data. Scores were grouped into perfect (25/25) vs. non-perfect to address ceiling effects, enabling logistic regression. Participant characteristics were categorized by role to assess associations with outcomes. |
| --- | --- | --- | --- | --- |
| Statistical methods | 12 | (*a*) Describe all statistical methods, including those used to control for confounding | 4-5 | We used logistic regression to identify factors associated with perfect evaluation scores (score = 25), while zero-truncated negative binomial regression was used to account for over-dispersion and truncation in non-perfect scores (scores <25). The multivariate models controlled for potential confounders, including type of IPE, profession, occupation, and AHP service. |
|  |  | (*b*) Describe any methods used to examine subgroups and interactions | 6 | Subgroup analyses were performed to explore differences in evaluation scores by type of IPE (facilitated discussion vs facilitated discussion with live consultation observation), profession (medical, nursing/care coordinator, or allied health), occupation (student or healthcare professional), and type of AHP service (dietetics, financial counselling, medical social services, physiotherapy, podiatry, psychology). Logistic regression models were used to identify significant associations within these subgroups. |
|  |  | (*c*) Explain how missing data were addressed | 4 | We collected all participants’ responses and there was no missing data. |
|  |  | (*d*) *Cross-sectional study*—If applicable, describe analytical methods taking account of sampling strategy | 5 | All available evaluation data from IPE sessions during the study period were included. No specific sampling strategy was applied, and analyses were conducted on the full dataset |
|  |  | € Describe any sensitivity analyses |  | No sensitivity analyses were performed for this study. |
| Results | | | | |
| Participants | 13* | (a) Report numbers of individuals at each stage of study—eg numbers potentially eligible, examined for eligibility, confirmed eligible, included in the study, completing follow-up, and analysed | 5 | A total of 352 participants attached to AHPs completed 1,168 IPE sessions with evaluation forms, which were included in the study. Of these, 667 (57%) were facilitated discussions and 501 (43%) included both facilitated discussions and live consultation observations |
|  |  | (b) Give reasons for non-participation at each stage | 8 | As this was a retrospective analysis of existing evaluation data, direct reasons for non-participation or nonresponse were not collected. However, it is plausible that factors such as scheduling conflicts, lack of interest, dissatisfaction with the IPE sessions, and clinical workload pressures may have contributed to some participants not completing evaluations. These potential influences may have introduced nonresponse bias in the study findings. |
|  |  | (c) Consider use of a flow diagram | 5 | Not applicable. This is a retrospective study analyzing existing evaluation data without participant recruitment or follow-up stages. Participant numbers are clearly described in the manuscript narrative. |
| Descriptive data | 14* | (a) Give characteristics of study participants (eg demographic, clinical, social) and information on exposures and potential confounders | 5 | Attendees' professional backgrounds were: 45.5% medical, 38.1% nursing or care coordinator, and 16.5% allied health. The majority (77.8%) were students. Medical social services accounted for the largest proportion of sessions (30.1%). The mean evaluation score was 23.7 (SD 2.1), with a median score of 25 (range: 15-25). Notably, 742 sessions (63.5%) received perfect scores (25/25). |
|  |  | (b) Indicate number of participants with missing data for each variable of interest |  | NA |
|  |  | (c) *Cohort study*—Summarise follow-up time (eg, average and total amount) |  | NA |
| Outcome data | 15* | *Cohort study*—Report numbers of outcome events or summary measures over time |  | NA |
|  |  | *Case-control study—*Report numbers in each exposure category, or summary measures of exposure |  | NA |
|  |  | *Cross-sectional study—*Report numbers of outcome events or summary measures | 5 | 352 participants attached to AHPs completed 1,168 IPE sessions with evaluation forms, which were included in the study. |
| Main results | 16 | (*a*) Give unadjusted estimates and, if applicable, confounder-adjusted estimates and their precision (eg, 95% confidence interval). Make clear which confounders were adjusted for and why they were included | 5-7 | Table 1.  Differences in Evaluation Scores between IPE modalities across services  Table 2.  Multivariate Hurdle Model Analyzing Factors Affecting Evaluation Scores for IPE |
|  |  | (*b*) Report category boundaries when continuous variables were categorized | 6-7 | Evaluation scores were categorized into perfect (score of 25) versus non-perfect (scores below 25) to facilitate logistic regression analysis. No other continuous variables, such as age, were collected or categorized in this study. |
|  |  | (*c*) If relevant, consider translating estimates of relative risk into absolute risk for a meaningful time period |  | NA |

Continued on next page

| Other analyses | 17 | Report other analyses done—eg analyses of subgroups and interactions, and sensitivity analyses | 6 | Table 2.  Multivariate Hurdle Model Analyzing Factors Affecting Evaluation Scores for IPE |
| --- | --- | --- | --- | --- |
| Discussion | | | | |
| Key results | 18 | Summarise key results with reference to study objectives | 7 | The study evaluated IPE modalities and found sessions with live consultation observations more likely to get perfect scores. Profession, occupation, and financial counselling attachment also predicted high scores. |
| Limitations | 19 | Discuss limitations of the study, taking into account sources of potential bias or imprecision. Discuss both direction and magnitude of any potential bias | 8 | The study has several limitations, including potential selection bias from non-randomized participant assignment and lack of demographic data, which limits analysis of influencing factors. Unmeasured variables such as prior experience could confound results. Use of Likert scales may introduce social desirability and facilitator likability bias. Self-reported evaluations collected anonymously immediately post-session might not reflect actual practice changes and could contribute to nonresponse bias if dissatisfied participants chose not to respond. |
| Interpretation | 20 | Give a cautious overall interpretation of results considering objectives, limitations, multiplicity of analyses, results from similar studies, and other relevant evidence | 7-8 | The findings suggest that IPE sessions with real-time experiential components are linked to higher perfect evaluation scores, likely due to increased engagement and relevance. Healthcare professionals rated sessions more favorably than students, reflecting differences in clinical experience and expectations. Variations across professional groups highlight the need to tailor IPE to diverse roles and experiences. While the statistical model addressed perfect score issues, ceiling effects and cultural biases may limit sensitivity to detect differences. Overall, results support incorporating authentic observation-based learning in IPE, especially for practicing professionals. |
| Generalisability | 21 | Discuss the generalisability (external validity) of the study results | 8 | Generalizability may be affected by the overrepresentation of medical students and the limited range of allied health professions |
| Other information | |  | | |
| Funding | 22 | Give the source of funding and the role of the funders for the present study and, if applicable, for the original study on which the present article is based | 9 | There was no funding. |

*Give information separately for cases and controls in case-control studies and, if applicable, for exposed and unexposed groups in cohort and cross-sectional studies.

**Note:** An Explanation and Elaboration article discusses each checklist item and gives methodological background and published examples of transparent reporting. The STROBE checklist is best used in conjunction with this article (freely available on the Web sites of PLoS Medicine at http://www.plosmedicine.org/, Annals of Internal Medicine at http://www.annals.org/, and Epidemiology at http://www.epidem.com/). Information on the STROBE Initiative is available at www.strobe-statement.org.
